# The methyltransferase METTL16 in digestive system cancers: functions and mechanisms

**DOI:** 10.3389/fonc.2026.1744928

**Published:** 2026-01-30

**Authors:** Jia Li, Yueyuan Bao, Fei Yao, Qingming Wu

**Affiliations:** 1 Institute of Infection Immunology and Tumor Microenvironment, School of Medicine, Wuhan University of Science and Technology, Wuhan, China; 2 Hubei Province Key Laboratory of Occupational Hazard Identification and Control, School of Medicine, Wuhan University of Science and Technology, Wuhan, China; 3 Scientific Research Office, Wuchang Hospital, Wuhan University of Science and Technology, Wuhan, China; 4 Department of Gastroenterology, Tianyou Hospital, Wuhan University of Science and Technology, Wuhan, China

**Keywords:** digestive system cancers, M6A, methyltransferase, METTL16, RNA modification

## Abstract

N6-methyladenosine (m6A) methylation, the most prevalent mRNA modification, affects RNA transcription, splicing, and stability. Methyltransferase-like 16 (METTL16), a novel m6A methyltransferase, regulates the expression of target mRNAs via m6A-mediated modifications. The methyltransferase domain of METTL16 is essential for its catalytic activity. In addition to acting as a methyltransferase, METTL16 can also facilitate mRNA translation in an m6A-independent manner, thus regulating cancer development and progression. Accumulating evidence has indicated that METTL16 plays a pivotal role in the progression of various cancers by regulating cell proliferation, apoptosis, metastasis, and resistance to chemotherapy. In this review, we provide a narrative review of the functions of METTL16 and summarize its oncogenic and tumor-suppressive functions as well as its underlying mechanisms in human digestive system cancers. However, further in-depth studies are required to validate these findings. By comprehensively summarizing the current literature on METTL16, we provide a theoretical basis for its application as a diagnostic and prognostic marker as well as a potential therapeutic target for digestive system cancers.

## 1 Introduction

Digestive system cancers rank among the most common malignant tumors, mainly include esophageal cancer, gastric cancer, colorectal cancer, liver cancer, pancreatic cancer, gallbladder cancer (1). Malignant tumors of the digestive system characterized by high incidence, high mortality and poor prognosis, the 5-year survival rate of patients is generally lower than that of other common cancers (2). Digestive system cancers are mainly treated with surgery, chemotherapy and radiotherapy, which early-stage symptoms are insidious, and often diagnosed at the middle or advanced stage, resulting in unsatisfactory treatment outcomes (3). Therefore, investigating the specific mechanisms of the occurrence and progression of digestive system cancers and identifying new therapeutic targets are crucial for improving the patients survival rate.

The occurrence and development mechanism of digestive system cancers involves changes in genetics, epigenetics and transcriptomics, among which epigenetic modifications have received extensive attention in recent years (4). Relevant studies have indicated the chemical modification of nucleotides plays important roles in regulating cancer-related biological processes (5). Over 170 chemical modifications in various RNAs have been identified (6), among which the N6-methyladenosine (m6A) modification is one of the most prevalent (7, 8). The m6A modification, first discovered in1974 (9), has been observed in yeast, plants, flies, bacteria, humans, and other mammals (10, 11). The m6A modification is a dynamic and reversible process catalyzed by m6A methyltransferases (writers), recognized by RNA-binding proteins (readers), and eliminated by demethylases (erasers) (12–14). Accumulating evidence indicates that the m6A modification regulates target gene expression by altering mRNA splicing, stability, translation, and export (15–17). Dysregulation of m6A participates in the malignant progression of digestive system cancers by regulating oncogenes or tumor suppressor genes (18–20).

Several studies have focused on human m6A writers, with the methyltransferase-like 3 (METTL3)/methyltransferase-like 14 (METTL14) complex being the earliest identified methyltransferase (18). This complex is formed in the cytoplasm and localized in the nucleus (19), where it is recruited to target sites with the aid of accessory proteins, such as WTAP, RBM15, VIRMA, and ZC3H13, to regulate cellular m6A levels (20, 21). Methyltransferase-like 16 (METTL16), a newly discovered RNA methyltransferase, has attracted considerable attention. METTL16 is a highly conserved protein that is distributed in the nucleus and cytoplasm (22, 23). It was initially believed to be a ribosomal RNA methyltransferase (24) and has been confirmed to catalyze m6A formation on many substrate RNAs, including methionine adenosyltransferase 2A (MAT2A) (25), U6 small nuclear RNA (U6 snRNA) (26), and other RNAs. Compared with the METTL3/METTL14 complex, METTL16 is a monomer with unique structures that can methylate double-stranded RNA and interact with multiple RNAs (27).

Regarding its function, numerous studies have demonstrated that METTL16 plays a key role in proliferation, invasion, metastasis, and drug resistance in digestive system cancers in an m6A-dependent or -independent manner (28, 29). In this review, we explored recent studies on METTL16 and digestive system cancers, and summarized the roles and underlying mechanisms of METTL16 in the occurrence and development of digestive system tumors, providing a theoretical basis for its application in cancer treatment.

## 2 METTL16 substrates

METTL16 has been reported to interact with mRNA and noncoding RNA (30), among which MAT2A, U6 snRNA, and MALAT1 have been the most extensively studied.

S-adenosylmethionine (SAM) is a primary cell methylation donor, and MAT2A encodes SAM synthetase in most human cells (31, 32). METTL16 binds to the hairpin structure of the 3′ UTR of MAT2A and modulates the splicing and expression of MAT2A mRNA, thereby regulating intracellular SAM levels (26, 31, 33). At elevated SAM concentrations, METTL16 briefly binds to the hairpin structure of hp1 for catalytic methylation and then rapidly dissociates, thereby preserving the intronic isoform of MAT2A mRNA. Conversely, low levels of SAM extend the residence time of METTL16 on hp1, stimulating the splicing of MAT2A, producing more mature MAT2A mRNA, and supplementing SAM levels (26).

METTL16 is a methyltransferase of U6 snRNA (34, 35). Pendleton et al. demonstrated that METTL16 methylates U6 snRNA at A43 by incubating purified recombinant METTL16 methyltransferase domain with radiolabeled U6 snRNA at position A43 (26). Aoyama et al. found that the C-terminal VCR increased the affinity and interaction of METTL16 with U6 snRNA. Specifically, the interaction between the VCR and the internal stem-loop within the U6 snRNA can induce a conformational rearrangement of U6 snRNA A43, altering the RNA structure to effectively facilitate the methylation of U6 snRNA by the methyltransferase domain (36). Taken together, these studies indicates that METTL16 is a pivotal RNA methyltransferase responsible for U6 snRNA methylation.

Metastasis-associated lung adenocarcinoma transcript 1 (MALAT1) is an oncogenic long non-coding RNA (lncRNA) involved in the development of various cancers (37–39). Brown et al. first demonstrated that METTL16 can specifically recognize and bind the 3′-terminal triple helix structure of MALAT1. However, the VCR domain of METTL16 was critical for this binding to occur (40). In addition, m6A-modified adenosine was found at position 8290 in MALAT1 near the METTL16-MALAT1 interaction site, although it appeared to have been deposited by METTL3/14 (41). Breger et al. found that the MALAT1 triple helix lacks the UACAGAGAA nonamer motif present in the U6 snRNA and MAT2A hairpins; therefore, the MALAT1 triple helix cannot be methylated by METTL16 (35). The purpose and function of METTL16 binding to the MALAT1 triple-helix complex are currently unknown, METTL16 may play a role in independent methylation.

## 3 The role and mechanism of METTL16 in human digestive system cancers

**Figure 1:**
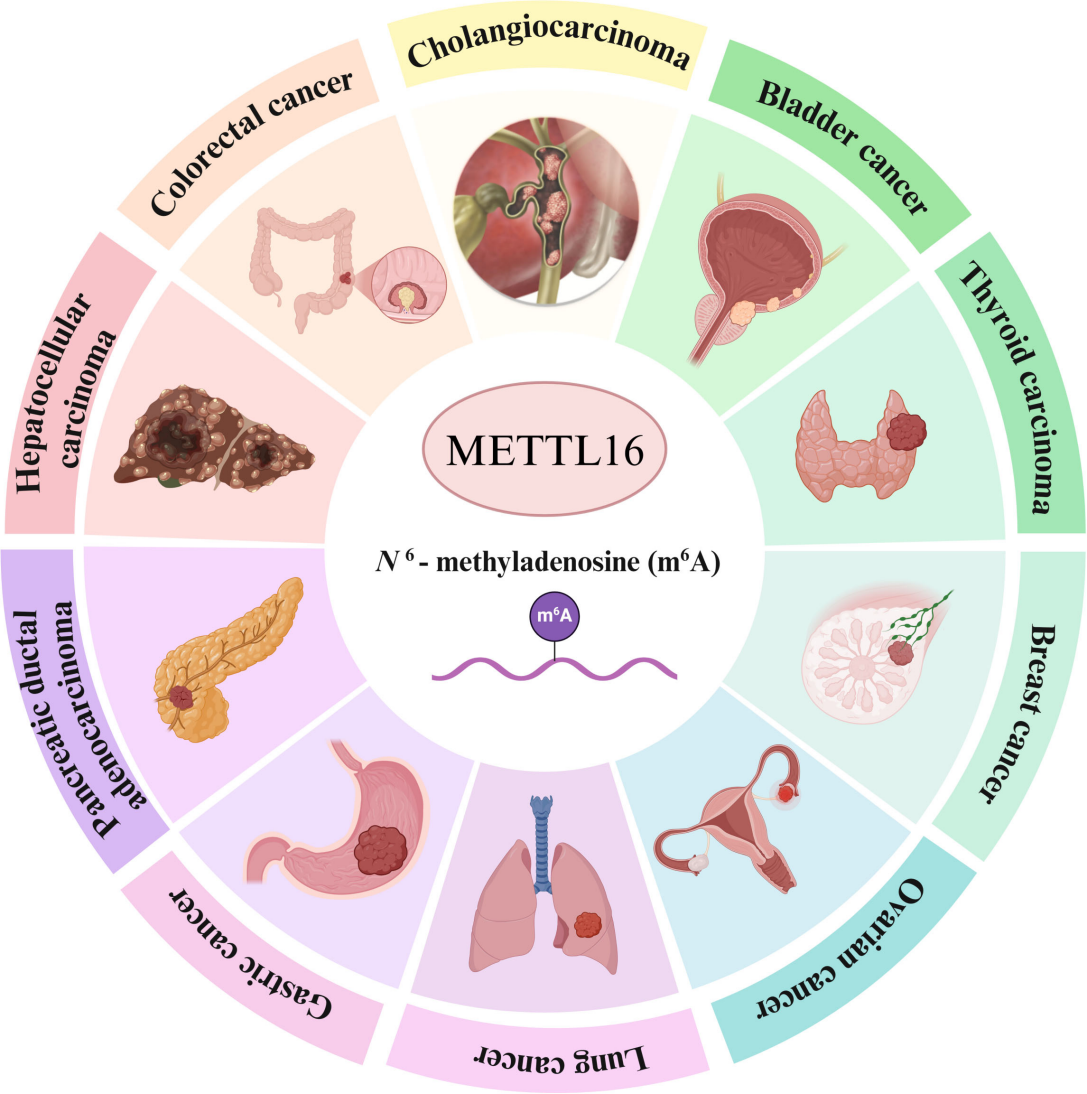
Schematic overview of METTL16 in human cancers, including cholangiocarcinoma, bladder cancer, thyroid carcinoma, breast cancer, ovarian cancer, lung cancer, gastric cancer, pancreatic ductal adenocarcinoma, hepatocellular carcinoma, and colorectal cancer.

METTL16 can regulate gene expressions through m6A-dependent and m6A-independent approach. On the one hand, it catalyzes the m6A modification of specific RNA substrates, and regulates the biological functions of tumor cells by altering mRNA splicing, stability, translation, and export. On the other hand, METTL16 directly interacts with eIF3a/b, significantly enhancing translation efficiency. The gain or loss of function mutations in METTL16 does not affect its interaction with eukaryotic translation initiation factor 3 a/b (eIF3a/b), indicating that the promotion of translation by METTL16 is independent. Many studies have shown that METTL16 plays an important role in the development and progression of human cancers (**Figure 1**)and could serve as a diagnostic and prognostic marker as well as a therapeutic target for cancers (42–44). We reviewed the roles and mechanisms (such as signalling pathways) of METTL16 in digestive system cancers (**Table 1**) to provide a theoretical basis for further research and clinical treatment.

**Table 1:** Role of METTL16 in digestive system cancers.

Cancer type	Expression	Target	Biological function	Mechanism	Ref
Hepatocellular carcinoma	Upregulated	LncRNA RAB11B-AS1	Promote cell migration, invasion, proliferation, and inhibit cell apoptosis	Reduce RAB11B-AS1 stability	(29)
		Lnc-CSMD1-7	Promote cell metastasis	Downregulate the Lnc-CSMD1–7 RNA stability to affect alternative splicing	(45)
		LncRNA TIALD	Promote cell EMT and metastasis	Reduced TIALD RNA stability to inhibit degradation of AURKA	(46)
		eIF3a	Accelerate CSC self-renewal and promote HCC progression	Interact with eIF3a	(47)
Colorectal cancer	Upregulated	SOGA1	Trigger mitotic progression	Enhance SOGA1 mRNA stability	(49)
			Promote metabolic reprogramming	Enhance SOGA1 mRNA stability to inhibit AMPK activity and upregulate PDK4	(42)
		TM7SF2	Promote lipid metabolic reprogramming	Upregulate TM7SF2 expression	(50)
Pancreatic ductal adenocarcinoma	Downregulated	p21	Suppress cell proliferation	Increase CDKN1A mRNA stability	(52)
		DVL2	Accelerate metastasis and invasion	Suppress DVL2 translation to inhibit Wnt/β-catenin signaling activation	(53)
		PD-L1	Participate in antitumor immunity	Regulate the tumor microenvironment and promote antitumor immunity	(54)
		MRE11	Inhibit DNA damage end-cleavage and cellular HR repair	Interact with MRE11 to repress its exonuclease activity	(55)
Gastric cancer	Upregulated	cyclin D1	Promote cell proliferation and tumor growth	Enhance cyclin D1 mRNA stability	(57)
		FDX1	Induce cuproptosis	Upregulate FDX1 mRNA stability	(58)
Cholangiocarcinoma	Upregulated	PRDM15	Promote cell proliferation and tumor progression	Increase translation of PRDM15 mRNA	(60)

### 3.1 Hepatocellular carcinoma

**Figure 2:**
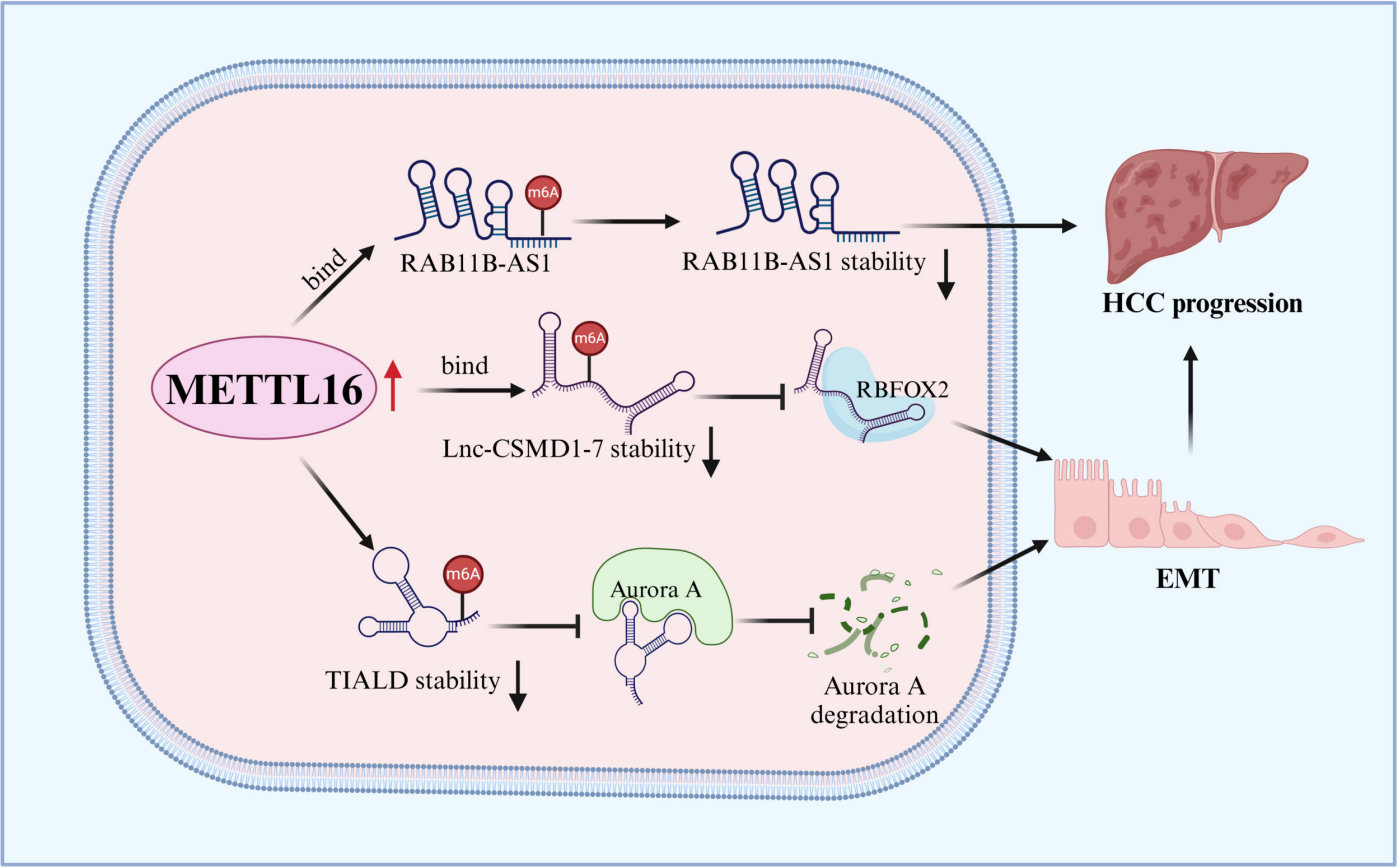
Schematic summary of the oncogenic roles of METTL16 in HCC. METTL16 promotes the progression of HCC by regulating the expression of RAB11B-AS1, Lnc-CSMD1–7 and TIALD in a m6A-dependent manner.

Liver cancer is the third leading cause of cancer-related deaths worldwide (2), among which hepatocellular carcinoma (HCC) is the most common type. METTL16 levels are elevated in HCC tissues and are correlated with poor patient prognosis. The overexpression of METTL16 promotes HCC cell migration, invasion, and proliferation and inhibits cell apoptosis, indicating its carcinogenic effects. Mechanistically, METTL16 directly binds to the tumor suppressor lncRNA RAB11B-AS1 and catalyzes m6A methylation, reducing its stability and expression. The overexpression of RAB11B-AS1 reverses the carcinogenic effects of METTL16 in HCC (29). METTL16 has also been reported to bind lnc-CSMD1-7, destabilizing it and inhibiting its binding to the splicing factor RNA binding fox-1 homolog 2 (RBFOX2), thereby promoting HCC cell migration (45). In addition, Wang et al. found that METTL16 downregulated lncRNA TIALD by catalyzing its m6A modification and reduced its RNA stability, thereby inhibiting the degradation of the oncogene aurora kinase A (AURKA) and ultimately promoting HCC migration and the epithelial-mesenchymal transition (EMT) process (46). Moreover, METTL16 is highly expressed in hepatocellular carcinoma stem cells (CSCs), where it promotes ribosomal RNA maturation and regulates mRNA translation by interacting with eIF3a, thereby accelerating the self-renewal of CSCs and promoting HCC progression (47). In summary, METTL16 plays an important role in the progression of HCC (**Figure 2**).

### 3.2 Colorectal cancer

Colorectal cancer (CRC) is a common malignancy of the digestive system that has the third-highest incidence rate and the second-highest mortality rate (48). METTL16 is highly expressed in CRC tissues compared to adjacent tissues and is significantly correlated with tumor size, lymph node metastasis, and tumor clinical grade (45). Mechanistically, Li et al. demonstrated that increased METTL16 promotes the m6A modification of autophagy associated 1 (SOGA1) mRNA, enhancing its mRNA stability in an insulin-like growth factor 2 mRNA-binding protein 1 (IGF2BP1)-dependent manner, triggering mitotic progression, and promoting CRC progression (49). In addition, Wei et al. also found that METTL16 could facilitate the m6A modification of SOGA1 mRNA. Subsequently, SOGA1 inhibits AMP-activated protein kinase (AMPK) activity and upregulates the expression of pyruvate dehydrogenase kinase 4 (PDK4), a key enzyme in glucose metabolism, thus promoting CRC metabolic reprogramming and progression (42). And the latest research supports that the highly expressed METTL16 promotes CRC progression and is related to the poor prognosis of patients (50), these studies indicate that METTL16, as a pro-carcinogenic factor, promotes the development and progression of CRC.

### 3.3 Pancreatic cancer

**Figure 3:**
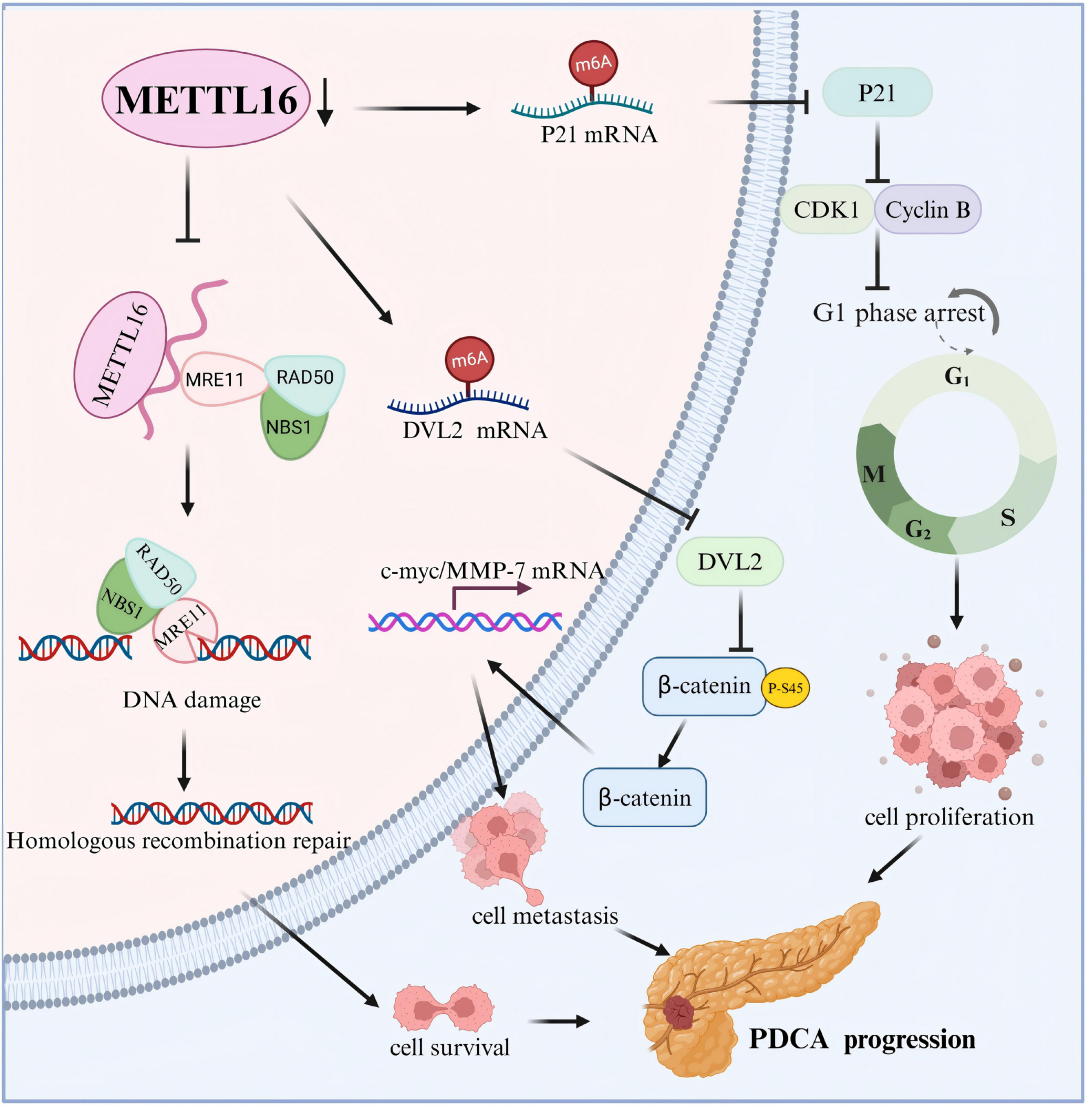
Schematic summary of the tumor-suppressive roles of METTL16 in PDAC. METTL16 represses the progression of PDAC by regulating the expression of p21 and DVL2 in a m6A-dependent manner and DNA damage repair in a m6A-independent manner.

Pancreatic ductal adenocarcinoma (PDAC) accounts for 85% to 90% of pancreatic cancers, with the most malignant and the poorest prognosis (51). It was reported that METTL16 is downregulated in PDAC, and its overexpression enhances the expression and stability of cyclin-dependent kinase inhibitor 1A (CDKN1A) (p21) through m6A modification, delaying the G1 phase transition and exerting a strong inhibitory effect on PDAC (52). Another study revealed a longer overall survival in patients with PDAC with higher METTL16 expression. METTL16 mediates the m6A modification of dishevelled segment polarity protein 2 (DVL2) mRNA, a key protein in the Wnt signaling pathway, suppressing the migration and invasion of PDAC cells by inhibiting the activation of the Wnt/β-catenin signaling pathway (53). Furthermore, Lu et al. demonstrated that METTL16 expression is markedly reduced in PDAC tissues and plays a crucial role in regulating the tumor microenvironment. Gain-of-function experiments indicated an abundance of native B and CD8+ T cells, a decrease in M0 macrophages, and reduced expression of the immune checkpoint PD-L1 in the high METTL16 expression group (54). Zeng et al. showed that METTL16 represses the exonuclease activity of meiotic recombination 11 (MRE11) in a methyltransferase-independent manner, inhibiting DNA terminal resection. METTL16 is phosphorylated following DNA damage, which leads to conformational changes in RNA binding and reduces the interaction between METTL16 and MRE11, thereby promoting damaged DNA cleavage and accelerating the cellular homologous recombination (HR) repair process of pancreatic ductal adenocarcinoma (55). However, the specific mechanism through which METTL16 regulates the PDAC tumor microenvironment remains unclear. Collectively, these findings indicate that METTL16 plays a tumor-suppressive role in PDAC and may be a therapeutic and prognostic biomarker (**Figure 3**).

### 3.4 Gastric cancer

**Figure 4:**
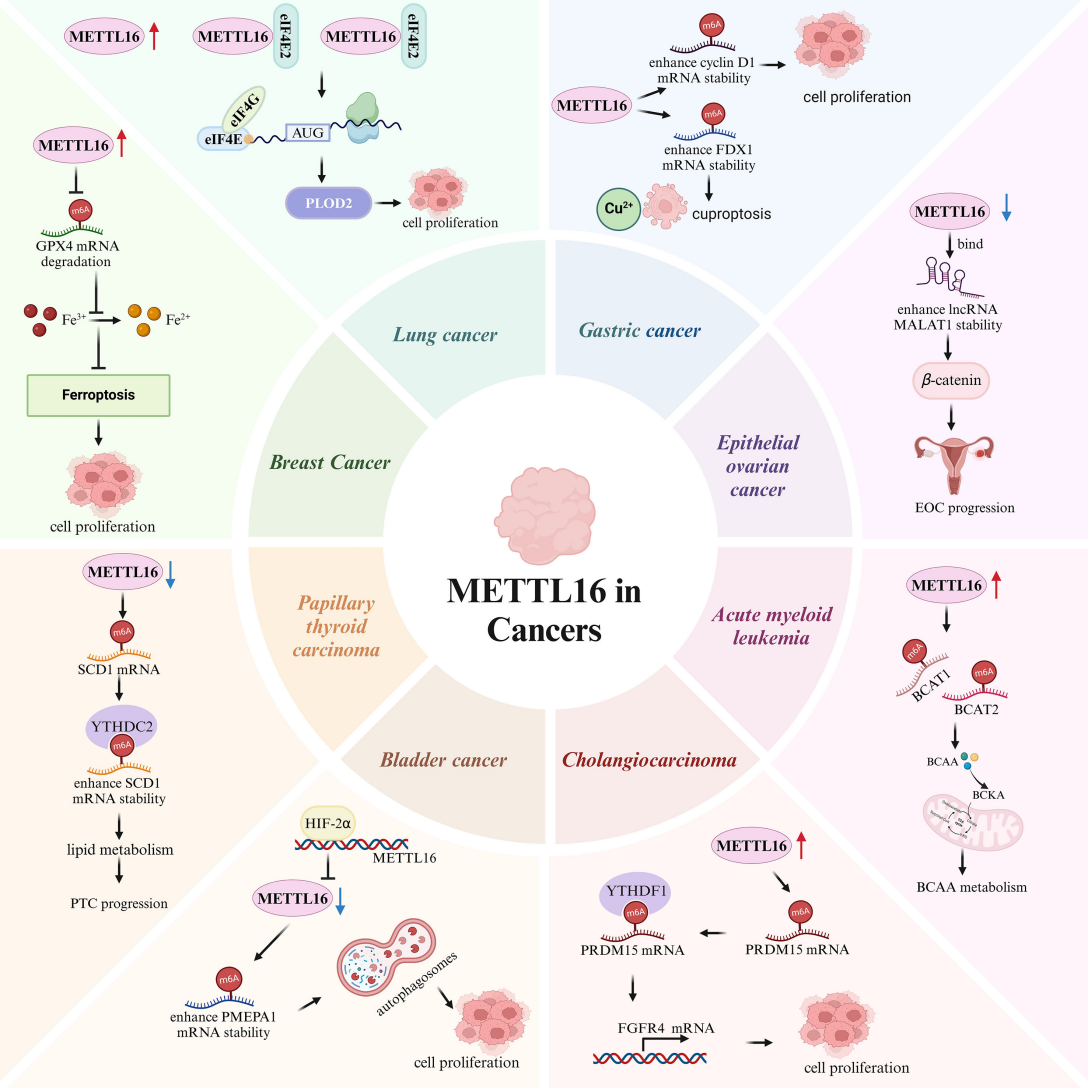
Schematic overview summarizing dysregulated METTL16 and representative m6A-related targets/pathways across multiple cancers (lung, breast, gastric, epithelial ovarian, acute myeloid leukemia, cholangiocarcinoma, bladder, and papillary thyroid carcinoma).

Gastric cancer (GC) ranks among the top five malignancies worldwide in both incidence and mortality (56). METTL16 is highly expressed in GC tissues compared to adjacent normal tissues, and its knockdown inhibits cell proliferation and induces cell-cycle arrest at the G1/S phase. Mechanistically, METTL16 enhances cyclin D1 mRNA stability through its m6A methyltransferase activity, increasing cyclin D1 expression and accelerating GC cell proliferation (57). Additionally, Sun et al. found that the lactylation of METTL16-K229 increases ferredoxin 1 (FDX1) expression, promoting the reduction of Cu^2+^ to the more toxic Cu^1+^ and thereby inducing cuproptosis in GC (58) (**Figure 4**). Collectively, current evidence suggests that METTL16 promotes GC progression through distinct mechanisms.

### 3.5 Cholangiocarcinoma

Cholangiocarcinoma (CCA) is a highly malignant bile duct tumor with an overall 5-year survival rate that remains below 20% (59). Liu et al. showed that METTL16 is upregulated in CCA and that its knockdown significantly inhibits CCA growth. Mechanistically, METTL16 increases PR domain containing 15 (PRDM15) translation in an m6A-dependent manner, which is recognized by the m6A reader protein YTH N6-methyladenosine RNA-binding protein 1 (YTHDF1), increasing fibroblast growth factor receptor 4 (FGFR4) expression and promoting extracellular signal-regulated kinase 1/2 (ERK1/2) and protein kinase B (AKT) phosphorylation at Ser473, thereby promoting tumorigenesis (60) (**Figure 4**).

Taken together, METTL16 has been found to play different roles in the pathogenesis and progression of digestive system cancers. Functional studies on METTL16 could help us better understand its biological behavior and provide new perspectives and strategies for its applications in cancer diagnosis and treatment.

## 4 The possible determinants of the role of METTL16

According to the studies in the previous section, METTL16 is reported to act as an oncogene in some cancers and as a tumor suppressor in others. Here we summarized the possible determinants of the role of METTL16.

METTL16 possess tissue-specific effects, and its RNA targets vary among different types of cancer or tissues. The modification of m6A in CRC, HCC and GC can enhance the expression of tumor-related genes and promote tumorigenesis, while in PDAC it can repress tumor-related genes expression, thereby suppressing tumor progression. In addition, METTL16 may cooperate with various m6A reader proteins to bind and modify different RNAs, the downstream target genes and pathways directly determine its regulatory functions. For example, METTL16 promoting tumor development by up-regulating the expression of SOGA1, which is crucial for maintaining mitosis and chromosomal stability in cancer cells (49). And it inhibits tumor cell proliferation by regulating the expression of p21, which is a key cell cycle inhibitor protein (52). And the tumor microenvironment is also an important factor influencing the function of METTL16. Specifically, a high-lactate environment can induce lactation modification of METTL16 and promote cuproptosis in gastric cancer cells (58), and phosphorylation modifications of METTL16 triggered by DNA damage can also alter its RNA binding ability (52). Additionally, the metabolic state of tumor cells (such as oxygen levels and SAM availability) may alter the functions of METTL16 (32, 45).

In summary, the roles of METTL16 in digestive system cancers are not fixed, which are determined by multiple factors, such as the availability of SAM, tissue-specific RNA targets, and the tumor microenvironment.

## 5 Conclusion and prospects

With the improvement in living standards and changes in dietary patterns, the incidence and mortality rates of common digestive system tumors have been increasing and have become the main components of cancer-related deaths. An increasing number of studies demonstrated that m6A modifications play important biological roles in the development and progression of digestive system cancers (61–63). In this review, we focused on METTL16, an important m6A modulator that affects various mRNA processes (28, 47, 64), and discussed that METTL16 plays a key role in the occurrence and progression of digestive system cancers through m6A-dependent and -independent manners.

METTL16 is closely associated with the proliferation, invasion, and migration of digestive system cancers, which might serve as a potential diagnostic and therapeutic biomarker (60, 65). However, the role of METTL16 in human digestive system cancers have not been widely studied, which mainly relies on cell models, bioinformatics analyses and immunodeficient mice (66), the existing studies on METTL16 have overlooked the existence of immune cells in the tumor microenvironment, the variability among datasets, and the lack of large-scale clinical validation studies. Targeting RNA methyltransferases has shown potential especially in cancer treatment, but it still faces multiple challenges. The specificity is the primary challenge, the recognition and modification of RNA sequences require high precision. If the targeting is inaccurate, it may affect normal gene functions and lead to off-target effects (67, 68). Additionally, METTL16 can directly promote mRNA translation in an m6A-independent manner, thus merely designing inhibitors targeting its catalytic pocket may not be able to completely block its cancer-promoting function, leading to treatment failure (28, 69, 70). Moreover, RNA methylation is involved in regulating gene expression and immune response processes. Therefore, inhibiting RNA methyltransferases enzymes may trigger cytotoxicity or immune responses (71–73). In addition, drugs targeting RNA methyltransferases (such as small molecule inhibitors) need to maintain sufficient concentrations in the body to target cancer tissues, but the intracellular delivery and bioavailability of RNA targets remain technical bottlenecks. Moreover, the complexity of the tumor microenvironment, hypoxia and metabolic reprogramming may weaken the efficacy of drugs (74–76). At present, except for a few METTL3 inhibitors that have entered preclinical research (77), most methyltransferases do not have effective targeted drugs and further in-depth research is needed.

In conclusion, METTL16 is abnormally expressed in digestive system cancers and acts as an oncogene or tumor suppressor that regulates cancer progression (**Table 1**). Although certain progress has been made in the research of METTL16, but most studies are based on cellular experiments and immunodeficient mice. Therefore, large-scale, multi-center clinical tumor sample studies are needed to verify the expression of METTL16 and explore its detailed mechanism and prognostic value. Taken together, a deeper understanding of the role and molecular mechanisms of METTL16 in human digestive system cancers will be of great significance for the early diagnosis, prognosis, and treatment of cancers.
